# Supplementary material for: 64-Channel Carbon Fiber Electrode Arrays for Chronic Electrophysiology
Source: Sci Rep. 2020 Mar 2;10:3830. doi: 10.1038/s41598-020-60873-y (PMC7052209; doi:10.1038/s41598-020-60873-y)
Supplement: Supplementary file 1 — Supplementary Information. [file 41598_2020_60873_MOESM1_ESM.pdf]

Supplementary Information

64-Channel Carbon Fiber Electrode Arrays for Chronic Electrophysiology

Grigori Guitchounts and David Cox

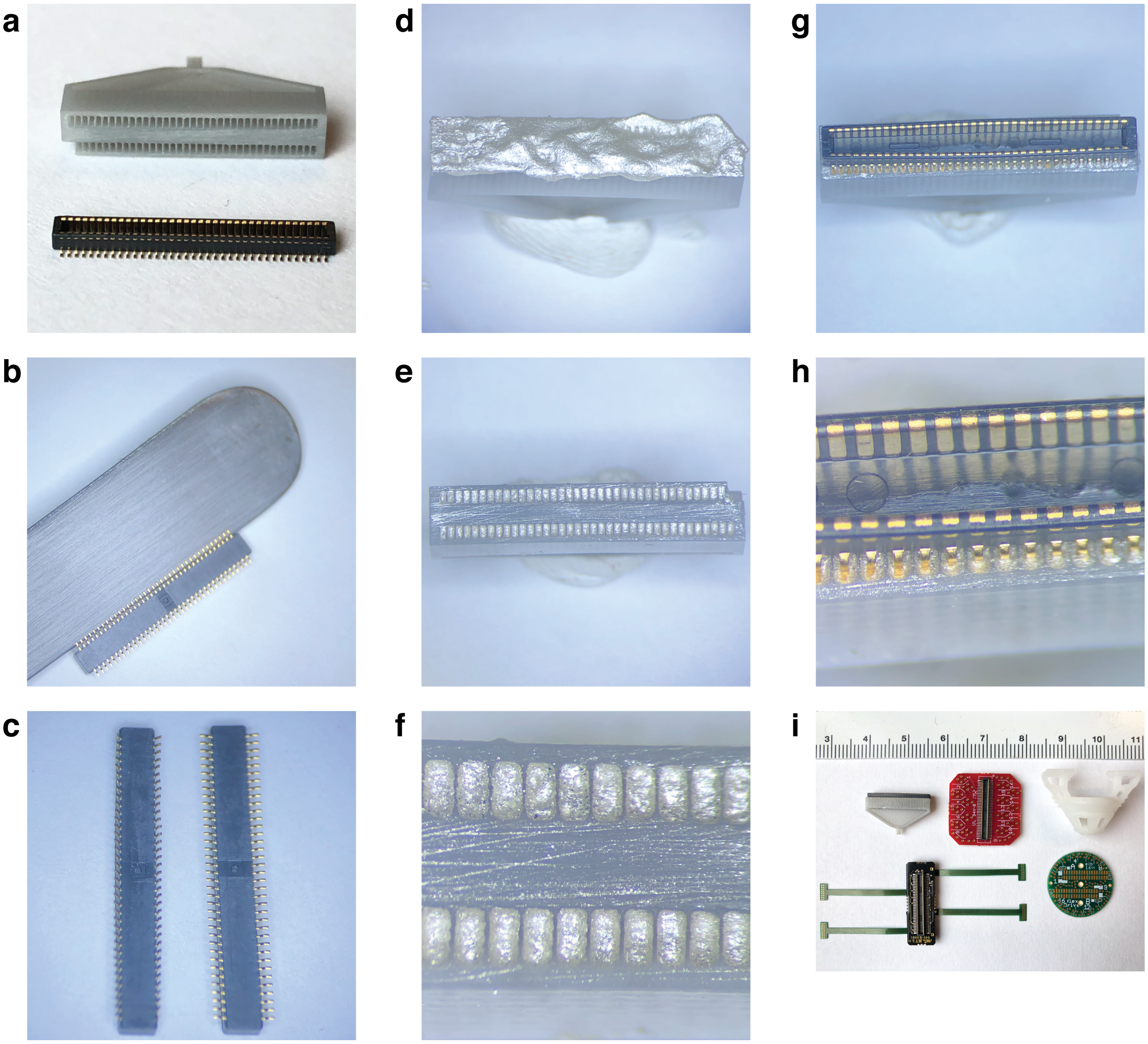

Figure S1. Details of Assembly Process. **(a)** Plastic block with Hirose connector before assembly. **(b)** The leads of the Hirose must be bent down 90 degrees so that they may fit into the wells of the plastic block. The blunt end of forceps, depicted here, may be used for this process. **(c)** A Hirose connector with leads bent down (left) next to one in its native state (right). **(d)** After carbon fibers are threaded through the plastic block and their ends freed from Parylene using a flame (see Guitchounts et al. 2013[27] for details), silver paint is used to electrically interface the fibers with the Hirose connector. Silver paint is dabbed on top of the plastic block and pushed into the wells with a wooden applicator or other flat surface (these samples were prepared without any carbon fibers, to facilitate the photography). **(e)** Once the silver paint has permeated the wells, excess paint is wiped off using a cotton-tipped applicator. **(f)** A close-up of the wells showing boundaries among them. **(g)** A plastic block with silver paint and a Hirose connector mounted manually into the wells. **(h)** A close-up of the bent Hirose leads extending into the wells of the plastic block. **(i)** A collection of arrays to demonstrate relative scale. Top row, from left to right: 64-channel plastic block and Hirose connector, an electrode interface board (EIB) with the same Hirose connector designed for tetrode arrays[53], flexDrive plastic block featuring 16 microdrive slots[55]; bottom row, left: PCBs to extend 64-channel CFEA for recordings in multiple brain regions. Four 16-channel flex cable PCBs are connected to a breakout PCB featuring a 70-pin Hirose connector that would mate with a headstage; right: EIB designed for use with the flexDrive[55].

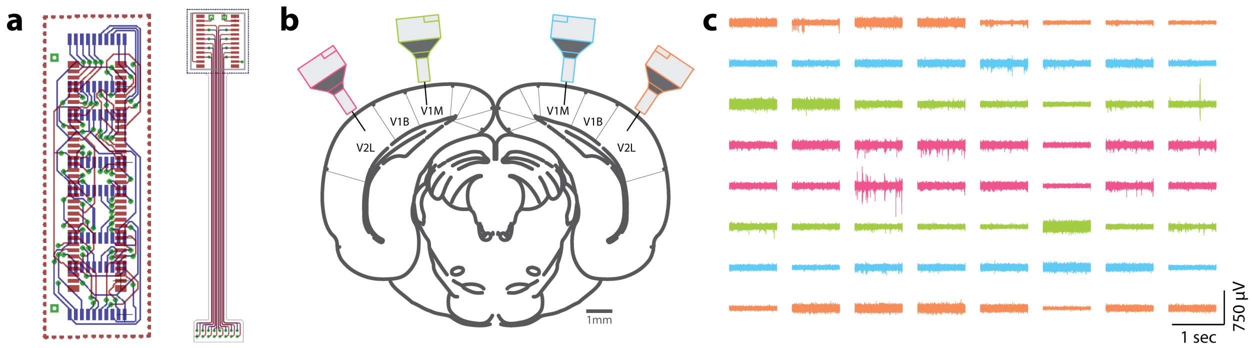

Figure S2. Multi-site Recordings. (a) Outlines of the PCBs for interfacing multiple recording sites. Left: breakout board to connect a headstage's 70-pin Hirose connector to four 20-pin Hirose connectors. Right: flex-PCB design which connects to the breakout board using a 20-pin Hirose connector (top), and to a plastic block housing electrode wires (bottom). **(b)** These devices were used to record from four different brain regions in one rat, targeting primary visual and extrastriate cortical areas. The cartoon coronal section of a rat's brain shows the locations the four arrays were implanted. The brain and arrays are depicted on the same scale. **(c)** Sample filtered traces showing multiunit activity recorded in the four regions, with colors of traces corresponding to the location of the arrays in **(b)**.

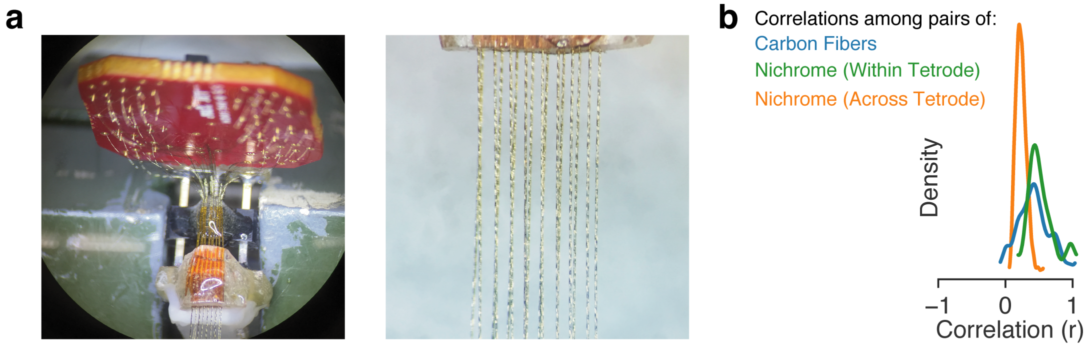

Figure S3. Cross-talk measurements. (a) Photographs of a 16-tetrode nichrome wire array, with tetrodes arranged in am 8x2 grid with ~300-micron spacing. **(b)** Correlation density among 30-second bandpass-filtered traces from pairs of wires in arrays implanted in rat V1. Correlations among pairs of nichrome wires within a given tetrode (green) tended to be higher than those among nichrome wires from different tetrodes (orange). Correlations among pairs of carbon fibers (blue) were slightly lower than those of wires within a tetrode. Means ± S.E.M.: CFEA: 0.42 ± 0.0068, Across Tetrode: 0.23 ± 0.0018, Within Tetrode: 0.54 ± 0.019.
